# No adverse effects of submelt-annealed highly crosslinked polyethylene in cemented cups: An RSA study of 8 patients 10 years after surgery

**DOI:** 10.3109/17453674.2011.652889

**Published:** 2012-04-24

**Authors:** Stephan M Röhrl, Bo Nivbrant, Kjell G Nilsson

**Affiliations:** ^1^Department of Orthopaedics, Oslo University Hospital, CIRRO, Oslo, Norway; ^2^Perth Orthopaedic Institute, University of Western Australia, Perth, Australia; ^3^Umeå University Hospital, Umeå, Sweden.

## Abstract

**Background and purpose:**

Highly crosslinked polyethylene (PE) is in standard use worldwide. Differences in the crosslinking procedure may affect the clinical performance. Experimenatal data from retrieved cups have shown free radicals and excessive wear of annealed highly crosslinked PE. We have previously reported low wear and good clinical performance after 6 years with this implant, and now report on the 10-year results.

**Patients and methods:**

In 8 patients, we measured wear of annealed highly crosslinked PE prospectively with radiostereometry after 10 years. Activity was assessed by UCLA activity score and a specifically designed activity score. Conventional radiographs were evaluated for osteolysis and clinical outcome by the Harris hip score (HHS).

**Results:**

The mean (95% CI) proximal head penetration for highly crosslinked PE after 10 years was 0.07 (–0.015 to 0.153) mm, and the 3D wear was 0.2 (0.026 to 0.36) mm. Without creep, proximal head penetration was 0.02 (–0.026 to 0.066) mm and for 3D penetration was 0.016 (–0.47 to 0.08) mm. This represents an annual proximal wear of less than 2 µm. All cups were clinically and radiographically stable but showed a tendency of increased rotation after 5 years.

**Interpretation:**

Wear for annealed highly crosslinked PE is extremely low up to 10 years. Free radicals do not affect mechanical performance or lead to clinically adverse effects. Creep stops after the first 6 months after implantation. Highly crosslinked PE is a true competitor of hard-on-hard bearings.

Highly crosslinked polyethylene (PE) has become a standard option in acetabular cups. Long-term results are still not available, since modern highly crosslinked PE (HXLPE) was introduced into the market around the shift of the millennium (Thomas et al. 2011). Fuelled by the debate about degradation through free radicals (Currier et al. 2007) and preservation of mechanical properties (Tower et al. 2007), there are at least 9 different highly crosslinked PEs with different production protocols commercially available. Although early pilot studies with HXLPE (Oonishi et al. 1998, Grobbelar et al. 1999. Wroblewski et al. 1999) with annealed or remelt-stabilized PE have shown good clinical results, modern first-generation HXLPE was introduced by McKellop (1999) and by Kurtz et al. (1999) in the late 1990s. Since then, second-generation HXLPE with either additives (vitamin E), mechanical enhancement (Kurtz et al. 2006a), or a sequential annealing process have been introduced and promise further improvement. Although HXLPE is in clinical use globally, little is known about the oxidative in vivo stability of these new polyethylenes (Muratoglu et al. 2010).

Concerns with annealed (non-remelted) HXLPE are free radicals trapped in the matrix, leading to degradation and excessive wear (Kurtz et al. 2006b, 2011). However, this is not supported by clinical data. So far, it appears that HXLPE reduces the risk of osteolysis (Digas et al. 2007, Jacobs et al. 2007, Callaghan et al. 2008, Kurtz et al. 2011). At the same time, alarming results with increased wear have been reported with remelted HXLPE from retrievals (Muratoglu et al. 2010).

We therefore measured femoral head penetration in a cohort with annealed first-generation highly crosslinked polyethylene with radiostereometry. Femoral head penetration is a substitute for in vivo wear measurement (Valstar et al. 2005, Bragdon et al. 2006). We consider it important to report on the 10-year wear measurement for 5 reasons: (1) we used RSA, a high-precision measuring method (Valstar et al. 2005), (2) retrieval studies have shown increased degradation of this HXLPE from oxidation (Currier et al 2007), (3) other HXLPEs have shown increasing wear after 5 years, (4) serum protein may influence wear performance negatively in the long term (St. John 2009), and (5) HXLPE is the most commonly used bearing material in THA worldwide. We therefore update our previous 6-year report on submelt-annealed crosslinked PE (Röhrl et al. 2005, 2007) with 10-year data on wear and clinical outcome.

## Patients and method

In 2000, 10 hips in 10 patients were operated consecutively with annealed highly crosslinked polyethylene cups. The patients had a mean age of 61 (49–79) years. We refer to Röhrl et al. (2005, 2007) for further information regarding the surgical technique and information about the patients.

The patients received an all-polyethylene Osteonics (Stryker) cup made of Crossfire PE. This PE of GUR 1050 is highly crosslinked by irradiation with 7.5 Mrad, heat-annealed below melting temperature (120°C), and finally sterilized by irradiation with 2.5 Mrad, all in an inert atmosphere. All cups were cemented (Palacos with gentamicin) and combined with Exeter femoral stems with 28-mm metal heads.

The patients were examined with RSA at 2 months after surgery and at 1, 2, 3, 4, 5, 6, and 10 years. Analyses of wear and migration were done with the UmRSA system (RSA Biomedical, Umeå, Sweden). In accordance with earlier measurements (Röhrl et al. 2005, 2007), wear was measured and expressed as the proximal head penetration. Initial creep during the wear-in period was eliminated by subtracting the mean of the early (2-month) measurement from the mean of the 10-year measurement in each patient.

Patients were assessed with Harris hip score before surgery, at 2 years, and at the last visit. Activity was evaluated with UCLA activity score and also from the patient’s subjective perception of activity rated as very low, low, normal, high, or very high at 10 years.

Conventional radiographs were taken postoperatively, and at 2, 5, and 7 years. In order to reduce radiation exposure at the last follow–up, conventional radiographs were taken only if the patient had clinical symptoms suggesting loosening. The percentage of the cup interface covered by a radiolucent line (RLL) more than 1 mm wide was assessed together with inclination and position of the cup. Before the 10-year follow-up, 1 patient had died from causes unrelated to the arthroplasty and with his prosthesis unrevised. 1 patient was critically ill and was not available for the follow-up. This left 8 patients for clinical, radiographic, and radiostereometric evaluation. All patients had good-quality radiographs. The precision for proximal wear was 0.09 mm, expressed as absolute mean penetration + 1.96 SD (Valstar et al. 2005). Table 1 shows the precision for all cardinal planes.

**Table 1.** Precision of wear, translation, and rotation

Axis	Translation (mm)	Rotation (°)	Wear
x	0.30	0.47	
y	0.39	0.49	0.09
z	0.19	0.22	
3D			0.31

The study was approved by the ethics committee of the University of Umeå, Sweden.

### Statistics

Changes in wear over time were assessed by Wilcoxon signed rank test (comparing longitudinal changes within the group). To eliminate creep, the 2-month penetration was subtracted for each patient individually. Changes over time were tested with related-samples Wilcoxon signed rank test.

## Results

### Wear

**Figure 1.**
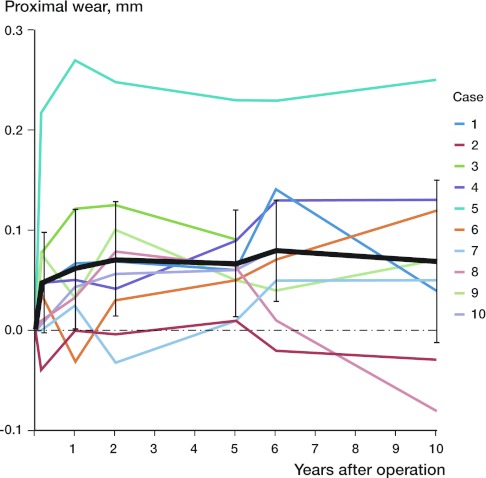
Proximal penetration/wear for the individual patients (colored lines) and the mean (black line) with 95% CI (whiskers).

**Figure 2.**
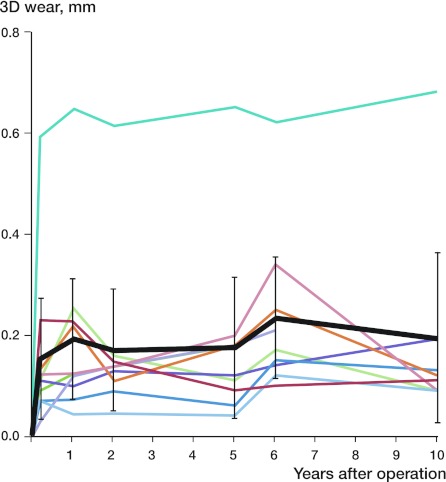
3-dimensional penetration/wear for the individual patients (colored lines) and the mean (black line) with 95% CI (whiskers).

The mean (95% CI) proximal head penetration for highly crosslinked PE after 10 years was 0.07 (–0.015 to 0.153) mm (Figure 1), whereas the 3D penetration was 0.2 (0.026–0.36) mm (Figure 2). Mean head penetration was 0.09 (–0.02 to 0.02) mm posterior and 0.07 (–0.087 to 0.22) mm medially. Subtracting the initial 2-month creep, the resulting mean proximal head penetration at 10 years was 0.02 (–0.026 to 0.066) mm and mean 3D wear was 0.016 (–0.47 to 0.08) mm. This corresponds to a proximal annual wear for crosslinked PE of less than 2 µm/year.

Considering the first 2–3 months mainly as wear, changes from 3 months onwards up to 10 years were not statistically significant for proximal head penetration (p = 0.3) or for total head penetration (p = 0.6).

### Cup stability

**Figure 3.**
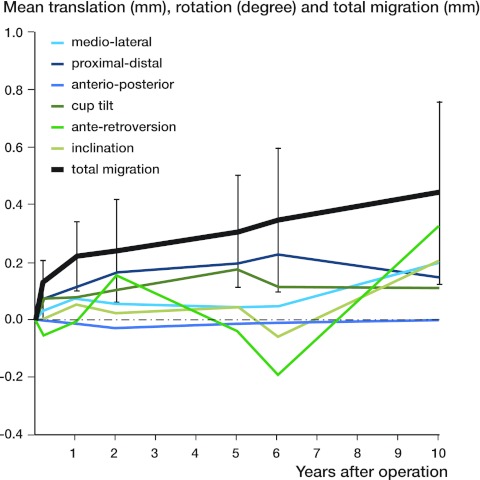
Mean total migration (black line) with 95% CI (whiskers) and mean translation/rotation in each plane for all cups up to 10 years. The cups were stable for translations, but we saw an increase in inclination after 7 years. In accordance with this, 3D motion increased after 7 years (black line). The change was not statistically significant.

The cups showed no statistically significant movement in any cardinal direction compared to the 2-month and 6-year positions (Figure 3). Mean 3D migration of all cups slightly increased after 7 years from 0.35 (0.097–0.598) to 0.44 (0.12–0.761) (p = 0.1).

### Clinical outcome

None of the 8 patients had pain in the operated hip. 5 patients participated regularly in activities such as bicycling, golf, or bowling (Table 2). 1 patient had low activity as a consequence of 3 cerebral strokes.

**Table 2.** Clinical data for all patients with clinical outcome (HHS, UCLA score, and individual perception of activity level). Patients 3 and 10 were excluded from the 10-year follow-up

A	B	C	D	E	F	G	H	I	J
1	male	69	Stroke x 3	70	51	44	76	3	low
2	female	67	no	70	49	44	91	6	low
3	male	89	Died		49				
4	male	66	no	62	54	44	100	8	normal
5	female	65	no	60	64	44	100	8	high
6	female	64	no	65	43	44	100	7	high
7	male	78	no	75	54	44	93	6	normal
8	female	70	no	87	51	44	98	7	normal
9	male	86	no	90	63	44	95	8	normal
10	male	59	Alcohol-related disease		28				
Total				72	50	44	96	6.3	
(SD)				(11)	(10)		(12 )	(1.6)	

A Patient no. B Sex C Age at follow-up D Complication E Weight (10 y) F Cup inclination G Pain (HHS) H HHS I UCLA J Activity perception

## Discussion

Since the introduction of highly crosslinked PE, there has been an ongoing discussion about whether the free radicals that are scarcely trapped in the annealed PE would lead to degeneration and cause inferior performance of the PE. Excessive wear of annealed HXLPE has been detected in various retrieval studies (Kurz et al. 2006b, Wannomae et al. 2006, Currier et al. 2007). In accordance with earlier clinical studies reporting on PE full of free radicals (Oonshii et al.1998), we found hardly any wear in the cemented cups with annealed highly crosslinked PE at 10 years. The clinical outcome was excellent.

The cups seemed to be stable when looking at the movement in each single plane, although the 3D migration increased slightly after 7 years. One explanation may be the biological reaction at the interface between the cement and the bone. As there is no direct ingrowth of bone into PE, this zone is slowly transformed into a fibrous layer (Schmalzried et al. 1992, Ryd et al. 1999). High wear has been a confounding factor when evaluating stability in cemented components with oxidation-prone conventional UHMWPE. It is possible that the interface is remodeled to a fibrous layer even without wear particles as a trigger. Another explanation might be methodological, as the stability of the segment (rigid body) defined by the markers within the acetabulum decreases over time. After 10 years, some of the tantalum balls might have moved slightly within the bone—leading to an increase in quantitative movement when reconstructing all planes. Clinically, there were no signs of loosening or failure.

Severe oxidation occurs about 1–3 mm beneath the surface. It was therefore predicted by Kurtz et al. (2006b) that when the surface layer has worn off after about 10 years, excessive wear will ensue. Our findings do not support this prediction. Most of the oxidation after implantation supposedly occurs in the non-bearing part of the cup, hence the rim. Kurtz et al. (2006b) also postulated that the weight-bearing part of the polyethylene is protected by the femoral head from oxidation. As long as this contact area has little wear, the cups perform well.

Increased fatigue crack propagation of remelted HXLPE compared to conventional UHMWPE was an early concern based on experimental data. So far, no detrimental results with HXLPE cups have been reported (Jacobs et al. 2007). Recent clinical data by Kurtz et al. (2011) and Capello et al. (2011) have suggested that there is no difference between remelted and annealed highly crosslinked polyethylene after 10 years. In a meta-analysis, Kuzyk et al. (2011) found reduced wear for highly crosslinked polyethylene but they could not conclude that this had an effect on revision rate.

Our measurements during the first postoperative year indicate that creep happens during the first postoperative months. After 3 months, penetration of the femoral head is almost negligible. After 1 year, no penetration is measurable up to 10 years. We have commented on this in our earlier publications (Röhrl et al. 2007). Anticipating low wear in the early postoperative phase because of low activity, we consider that our measurements adequately describe creep of annealed highly crosslinked PE. Our findings are similar to those of Glyn-Jones et al. (2008). 1 patient (no. 5 in Figures 1 and 2) showed more proximal head penetration for all measurements, compared to the others. The penetration did not increase with time, however. The exact reason for this is unclear, but possible explanations are joint laxity or interposed soft tissue at the postoperative RSA radiograph.

We had no control group. Compared to earlier long-term reports in the literature on conventional PE (Sochart 1999), we found a substantially reduced wear rate. There have been few long-term RSA studies of the acetabular articulation. We measured a wear rate of 0.09 mm after 10 years, but this was for conventional PE with 32 ceramic heads (Röhrl et al. 2006). Dahl et al. (2011) found an annual wear rate of 0.096 mm with conventional PE and a 28-mm CoCr head. We found that wear of submelt-annealed HXLPE was far lower, with hardly any wear measurable over a 10-year period.

We studied only 8 patients. However, we found no cases of measurable wear even though we used a high-precision measuring method. The width of the confidence interval of wear without creep over a period of 10 years (0.04) was extremely low. The penetration of the femoral head was below the precision of our method (90 μm). Thus, the upper limits of the confidence interval of changes observed after 10 years of wear were, for all clinical purposes, negligible.

Highly crosslinked PE appears to be a good alternative to hard-on-hard bearings, considering that metal-on-metal articulations may cause excessive wear and pseudotumors, and that ALVAL (Carothers et al. 2010, Schmalzried and Tiberi 2010) and ceramic-on-ceramic articulations have been plagued by squeaking (Sexton et al. 2011).

The third generation of HXLPE eliminates free radicals further either by mechanical compression, addition of antioxidants (vitamin E), or reiterated annealing. This might be a further step in conserving the excellent mechanical properties of HXLPE over a patient’s lifetime. However, we advise caution in extrapolating our results blindly to those newer brands of HXLPE. It is not clear whether these alterations might have a negative influence on the mechanical stability of the PE.
